# Cortical Plasticity Induced by Transcranial Magnetic Stimulation during Wakefulness Affects Electroencephalogram Activity during Sleep

**DOI:** 10.1371/journal.pone.0002483

**Published:** 2008-06-25

**Authors:** Luigi De Gennaro, Fabiana Fratello, Cristina Marzano, Fabio Moroni, Giuseppe Curcio, Daniela Tempesta, Maria Concetta Pellicciari, Cornelia Pirulli, Michele Ferrara, Paolo Maria Rossini

**Affiliations:** 1 Department of Psychology, University of Rome “La Sapienza”, Rome, Italy; 2 IRCCS Centro S. Giovanni di Dio, Hosp. Fatebenefratelli, Brescia, Italy; 3 Casa di Cura S. Raffaele Cassino and IRCCS S. Raffaele Pisana, Roma, Italy; 4 Laboratory of Sleep Psychophysiology, Faculty of Psychology, University of L'Aquila, L'Aquila, Italy; 5 Department of Internal Medicine and Public Health, University of L'Aquila, L'Aquila, Italy; 6 AFaR, Department of Neuroscience, Hosp. Fatebenefratelli, Isola Tiberina, Rome, Italy; 7 Neurology Clinic, University Campus Biomedico, Rome, Italy; University of Southern California, United States of America

## Abstract

**Background:**

Sleep electroencephalogram (EEG) brain oscillations in the low-frequency range show local signs of homeostatic regulation after learning. Such increases and decreases of slow wave activity are limited to the cortical regions involved in specific task performance during wakefulness. Here, we test the hypothesis that reorganization of motor cortex produced by long-term potentiation (LTP) affects EEG activity of this brain area during subsequent sleep.

**Methodology/Principal Findings:**

By pairing median nerve stimulation with transcranial magnetic stimulation over the contralateral motor cortex, one can potentiate the motor output, which is presumed to reflect plasticity of the neural circuitry. This paired associative stimulation increases M1 cortical excitability at interstimulus intervals of 25 ms. We compared the scalp distribution of sleep EEG power following paired associative stimulation at 25 ms to that following a control paradigm with 50 ms intervals. It is shown that the experimental manipulation by paired associative stimulation at 25 ms induces a 48% increase in amplitude of motor evoked potentials. This LTP-like potentiation, induced during waking, affects delta and theta EEG power in both REM and non-REM sleep, measured during the following night. Slow-wave activity increases in some frontal and prefrontal derivations and decreases at sites neighboring and contralateral to the stimulated motor cortex. The magnitude of increased amplitudes of motor evoked potentials by the paired associative stimulation at 25 ms predicts enhancements of slow-wave activity in prefrontal regions.

**Conclusions/Significance:**

An LTP-like paradigm, presumably inducing increased synaptic strength, leads to changes in local sleep regulation, as indexed by EEG slow-wave activity. Enhancement and depression of slow-wave activity are interpreted in terms of a simultaneous activation of both excitatory and inhibitory circuits consequent to the paired associative stimulation at 25 ms.

## Introduction

According to the two-process model of sleep regulation, the timing of sleep and wakefulness is modulated by the interaction of a homeostatic process, “Process S”, and by a circadian process, “Process C” [1]. Well established evidence on the homeostatic regulation of sleep suggests that slow-wave activity, i.e. EEG power in the 0.50–4.75 Hz range, depends on the duration of previous sleep and wakefulness, representing a marker of non-REM (NREM) sleep intensity [2]. This feature of sleep has consistently been shown in a broad range of species, including humans, cats, mice, rats, and squirrels [3]. Manipulations of sleep intensity by means, for example, of sleep deprivation, lead to clear homeostatic recovery processes [2] which do not involve the whole cerebral cortex to the same extent. Indeed, these recovery processes are mainly local and involve restricted brain regions, as shown in dolphins [4], birds [5], mice [6], rats [7], and humans [8], [9]. However, the neural mechanisms underlying the local changes of slow-wave activity (SWA) following increased sleep pressure are still unknown.

Recently, it has been hypothesized a link between local SWA and synaptic plasticity [10]. Plastic changes in local cortical circuits triggered by experience-dependent modifications during wakefulness should locally affect SWA during subsequent sleep. Consequently, this hypothesis predicts that increased synaptic strength after learning in wakefulness leads to a more synchronized EEG activity in subsequent sleep, as indexed by local increases of SWA. On the other hand, synaptic depression is associated to decreases of SWA [10]. The part of this hypothesis concerning the link between experience-dependent plasticity during wakefulness and local changes of SWA received a robust empirical support. Learning a visuomotor task [11] and localized potentiation of cortical responses evoked by transcranial magnetic stimulation during wakefulness [12] induce a local increase of SWA. On the contrary, the immobilization of a subject's arm produces a local decrease of SWA [13]. In these experiments, the size of the changes that were induced during waking also correlated to that of modifications in SWA, supporting the hypothesis that SWA during sleep is affected by plastic changes in local cortical circuits during wakefulness [13]. According to the hypothesis, these plastic changes should be associated to synaptic potentiation of specific cortical circuits, and should be dependent on long-term potentiation (LTP) mechanisms.

The introduction of a protocol with transcranial magnetic stimulation that induces LTP-like changes in human motor cortex allows a direct test of this prediction. Paired associative stimulation induces plastic cortical changes in sensorimotor cortex by coupling a single electric stimulus, delivered on a peripheral nerve, with a single magnetic pulse delivered on the scalp. The direction of cortical plasticity induced by paired stimulation depends on the interval between the stimuli. If sensory input from median nerve stimulation reaches the motor cortex at appropriate intervals prior to magnetic stimulation, paired associative stimulation potentiates local cortical excitability. Conversely, if magnetic stimulation precedes the arrival of sensory input to the cortex, paired associative stimulation causes a reduction of cortical excitability [14]–[17].

These plastic changes depend on a temporally asymmetric Hebbian rule [18]. As first demonstrated by Mariorenzi et al. [19], a short inter-stimulus interval at 10 ms produces a decrease in the motor evoked potential, measured after delivering a single magnetic pulse. On the other hand, a longer interval at 25 ms induces an increase in motor evoked potentials [16], [20]. A 10-ms interval allows to the sensory stimulation to reach the cortical relays, while a 25-ms interval is a sufficient time for the afferent stimulus to reach the contralateral sensorimotor cortex. Longer intervals (from 35 to 50 ms) do not affect motor evoked potentials [16].

Excitability changes related to paired associative stimulation at 25 ms evolve rapidly (within 30 min), are long lasting (at least 60 min), reversible and cortically generated [14]. Considering all these evidences, an LTP-like mechanism mediating the effects to paired associative stimulation was proposed [14], [16], [21].

**Figure 1:**
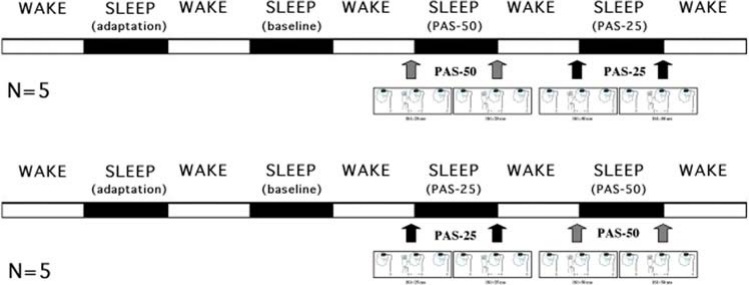
Experimental protocol. Sleep recordings were scheduled in the first night (adaptation), in the second night (baseline sleep), and in the third and fourth nights (post-paired associative stimulation sleep). In the third or fourth night subjects were submitted to a paired associative stimulation paradigm with a 25-ms interval (PAS-25), in the one night, and to a PAS paradigm with a 50-ms interval (PAS-50), in the other. The sequence of PAS-25 and PAS-50 was counterbalanced between subjects. Subjects were then retested after the final morning awakenings.

Accordingly, a potentiation of synaptic transmission in motor cortex, induced using an appropriately timed magnetic stimulation during the wake period, should coherently affect SWA in specific cortical circuits during subsequent sleep. The present study is aimed to compare the effects on sleep EEG topography of two types of paired associated stimulation: an LTP-like paradigm inducing synaptic potentiation, and a control condition, which does not affect synaptic potentiation. Specifically, LTP-like changes were induced by a paired associative stimulation at 25 ms, while the control condition was represented by a paired associative stimulation at 50 ms. It was hypothesized that only the paradigm with stimulation at 25 ms would induce a localized enhancement of SWA. To this aim, scalp EEG recordings of healthy subjects were studied in four consecutive sleep nights; subjects underwent a protocol with paired associative stimulation at 25 ms or at 50 ms (PAS-25 and PAS-50, respectively) before going to sleep in the third and fourth nights (a diagram of the complete experimental protocol is reported in Figure 1).

## Results

### Transcranial magnetic stimulation measures

#### Motor thresholds

The *Treatment* (pre-PAS vs. post-PAS) × *Night* (PAS-25 vs. PAS-50) analysis of variance on resting motor thresholds did not yield any significant main effect or interaction (F<1). Hence, motor thresholds did not change after the intervention with paired associative stimulation and were stable across the two experimental conditions (pre PAS-25 = 40.8%±2.1%; post PAS-25 = 40.5%±2.2%; pre PAS-50 = 40.9%±2.0%; post PAS-50 = 40.8%±2.1%).

#### PAS

**Figure 2:**
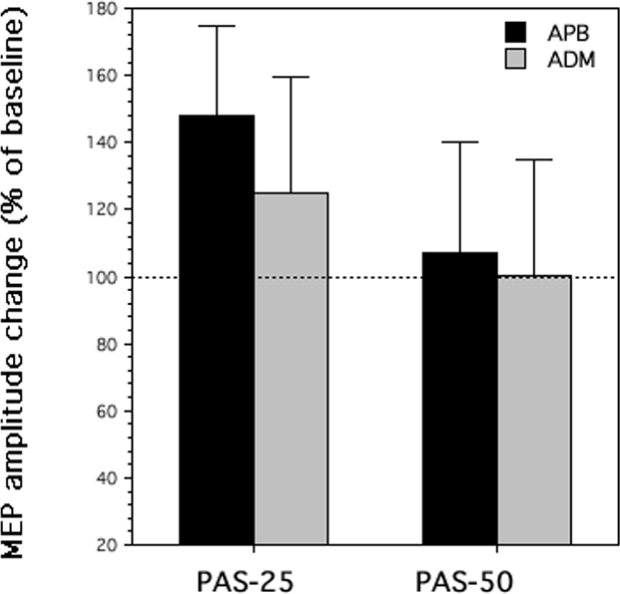
Effect of interval (PAS ) on motor evoked potential (MEP) amplitude recorded in in the right *abductor pollicis brevis* (APB) with a 25-ms (PAS-25) and a 50-ms interval (PAS-50), and in the right *abductor digiti minimi* (ADM) muscles. Data are expressed as a percentage of motor evoked potential (MEP) amplitude after PAS/before PAS. Zero corresponds to the MEP amplitude before PAS.

Results confirm the effectiveness of the experimental manipulation, because of a significant effect of *Treatment* on amplitudes of motor evoked potentials recorded from the right *abductor pollicis brevis* (F_1,9_ = 10.54; p = 0.01) and a significant *Treatment* × *Night* interaction (F_1,9_ = 11.68; p = 0.007). Therefore, the increase was limited to the PAS-25 intervention (p = 0.0006 at the post-hoc test), while the PAS-50 intervention did not affect motor evoked potential amplitudes (p = 0.79 at the post-hoc test). The paired associative stimuli at a 25-ms intervals enhanced motor evoked potentials to an extent of 48.13%±8.38 (Figure 2).

Results also confirm the specificity of the experimental manipulation, since the same analysis of variance on motor evoked potential amplitudes of *abductor digiti minimi* muscle –another hand muscle, but not involved by the conditioning stimulation on the median nerve- yielded no significant effect for *Treatment* (F_1,9_ = 0.69; p = 0.43) or *Treatment* × *Night* interaction (F_1,9_ = 2.86; p = 0.12).

### EEG measures

#### Polysomnography

##### Polysomnographic measures

Table 1 reports the results of the analyses of variance on polysomnographic variables. Comparisons between the nights do not show significant changes for any considered sleep variable. Hence, sleep architecture was not affected by generalized and nonspecific effects of transcranial magnetic stimulation (PAS-25 or PAS-50 vs. baseline) or by differential effects of the paired associative stimulation paradigms (PAS-25 vs. PAS-50).

**Table 1:** Means and standard errors of the polysomnographic variables, during the baseline and the experimental nights.

Sleep variables	Baseline		PAS-25		PAS-50		F (2,9)	P
	Mean	S.E.	Mean	S.E.	Mean	S.E.		
SL (min)	8.20	2.64	5.87	1.45	11.00	4.82	1.30	0.30
REM latency (min)	88.20	12.50	76.10	7.32	89.87	9.97	0.81	0.46
Stage 1 (%)	4.74	0.55	3.96	0.7	4.20	0.90	1.55	0.24
Stage 2 (%)	61.11	2.54	63.42	1.49	60.50	1.76	1.27	0.30
SWS (%)	9.49	1.84	7.61	1.62	9.36	1.97	1.78	0.15
NREM (%)	70.59	1.73	71.04	1.07	69.86	1.45	0.28	0.76
REM (%)	24.66	1.79	25.02	1.21	26.14	1.26	0.73	0.50
WASO (%)	5.16	1.26	3.99	0.89	3.41	1.08	2.69	0.09
Awakenings (#)	23.5	3.52	25.2	6.15	22.4	4.44	0.40	0.67
*Arousal* (%)	8.22	1.1	9.12	1.52	8.47	1.29	0.37	0.69
TST (min)	459.47	5.14	455.03	3.26	454.63	4.71	0.60	0.56
TBT (min)	492.43	9.60	480.54	6.37	481.37	9.81	1.67	0.22
SEI (TST/TBT)	0.94	0.01	0.95	0.008	0.95	0.01	0.83	0.45

The results of the one-way analyses of variance on log-transformed values are also reported.

SL = sleep latency; SWS = slow-wave sleep; REM = rapid eye movement sleep; WASO = wakefulness after sleep onset; TST = total sleep time; TBT = total bed time; SEI = sleep efficiency index.

#### Quantitative analysis of EEG

**Figure 3:**
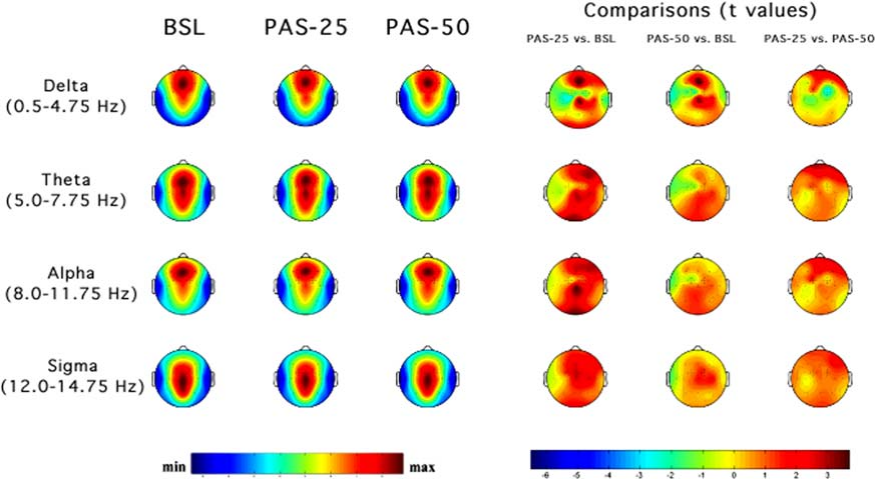
Topographic distribution of EEG power during NREM sleep. Average values were normalized by total power, colour coded, plotted at the corresponding position on the planar projection of the scalp surface, and interpolated (biharmonic spline) between electrodes. The maps are based on the 19 unipolar EEG derivations of the international 10–20 system with averaged mastoid reference, and on 10 additional derivations, positioned in both hemispheres over the left *individual* optimal site of transcranial magnetic stimulation and in each orthogonal direction at a distance of 1.5 cm from the hotspot (electrode positions indicated by dots). From the left, the first three columns show the absolute EEG power during NREM sleep of the baseline night (BSL), of the night preceded by the PAS-25 protocol (PAS-25), and of the night preceded by the PAS-50 protocol (PAS-50). Each row reports maps of EEG power in the delta, theta, alpha and sigma frequency ranges. To optimize contrast, each map was scaled separately between minimal (min) and maximal (max) power values. In the other three columns, the statistical maps (*t-values* maps) of the comparisons between PAS-25 vs. BSL, PAS-50 vs. BSL, PAS-25 vs. PAS-50 are illustrated for each frequency band (positive t-values indicate a prevalence of the first over the second term). Please note that, for the three columns on the right side, the colour code reports the actual *t-values* (the two-tailed level of significance of *p* = 0.02, after the Bonferroni correction, corresponds to a *t* = 2.82).

**Figure 4:**
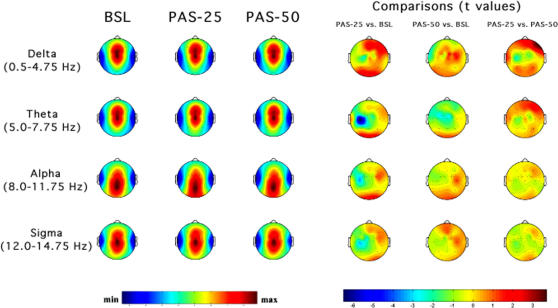
Topographic distribution of EEG power during REM sleep. Average values were normalized by total power, colour coded, plotted at the corresponding position on the planar projection of the scalp surface, and interpolated (biharmonic spline) between electrodes. The maps are based on the 19 unipolar EEG derivations of the international 10–20 system with averaged mastoid reference, and on 10 additional derivations, positioned in both hemispheres over the left *individual* optimal site of transcranial magnetic stimulation stimulation and in each orthogonal direction at a distance of 1.5 cm from the hotspot (electrode positions indicated by dots). From the left, the first three columns show the absolute EEG power during REM sleep of the baseline night (BSL), of the night preceded by the protocol of paired associative stimulation paradigm with a 25-ms interval (PAS-25) and of the night preceded by the protocol with a 50-ms interval (PAS-50). Each row reports maps of EEG power in the delta, theta, alpha and sigma frequency ranges. To optimize contrast, each map was scaled separately between minimal (min) and maximal (max) power values. In the other three columns, the statistical maps (*t-values* maps) of the comparisons between PAS-25 vs. BSL, PAS-50 vs. BSL, PAS-25 vs. PAS-50 are illustrated for each frequency band (positive t-values indicate a prevalence of the first over the second term). Please note that, for the three columns on the right side, the colour code reports the actual *t-values* (the two-tailed level of significance of *p* = 0.02, after the Bonferroni correction, corresponds to a *t* = 2.82).

Topographic distributions of EEG power during NREM and REM sleep for the selected frequency bands are illustrated in Figures 3 and 4 respectively. The figures depict EEG power maps for each frequency band, scaled between the maximum and minimum values, in the baseline night and in the two nights preceded by transcranial magnetic stimulation; maps of statistical differences between nights are also reported.

#### NREM sleep

An inspection of the power maps during baseline night reveals stable patterns within different frequency bands, and the data of maxima and minima exhibited the typical features of power spectra during NREM sleep. The delta and alpha bands exhibit a frontal midline predominance of power and minimum values over the temporal regions. In the theta band, the highest values are seen at the fronto-central midline areas, while the sigma band shows centro-parietal maxima. This frequency-specific regional pattern is roughly maintained in PAS-25 and PAS-50 nights.

The statistical maps on the right side of Fig. 3 reveal significant changes in the delta, theta, and alpha bands (two-tailed p = 0.02 corresponds to a t = 2.82). Both the protocols with transcranial magnetic stimulation (comparisons between baseline vs. PAS-25 or PAS-50 nights) enhanced delta power in both PAS-25 and PAS-50, with similar EEG power topography: significant increases were found at Fz and Cz scalp locations. Although not significant after correcting for multiple comparisons (p>0.02 and <0.05), some centro-frontal scalp locations close to the stimulated left motor cortex show a decreased delta power compared to the baseline night. The remaining significant changes were limited to baseline vs. PAS-25 comparisons: during PAS-25 night, theta power increased at the occipital sites (O1 and 02) and F4, while alpha power increased at the occipital sites (O1 and 02) and Cz. Hence, regardless of specific LTP-like changes, both the protocols with transcranial magnetic stimulation affected EEG power maps enhancing the delta and theta activity of centro-frontal areas; the occipital region also exhibits a significant increase of theta and alpha power in the PAS-25 condition.

More crucial for the aims of this experiment, the comparisons between PAS-25 vs. PAS-50 nights detail the pattern of EEG changes associated to synaptic potentiation. Significant changes affected the delta and theta bands, although the general topographic pattern is coherent for the alpha band. PAS-25 was associated with an enhanced delta and theta activity at some right frontal scalp locations (Fp2 and F8 for the delta band, and Fp1, Fp2, and F3 for the theta band) as compared to PAS-50. The enhancement of delta power at these frontal areas coexists with a specific topographical pattern of decreased activity in areas ipsilateral and contralateral to the hotspot of maximal motor cortex excitability (significant for RMa).

#### REM sleep

An inspection of the power maps in Fig. 4 reveals stable patterns within different frequency bands. In baseline sleep, the delta and theta bands exhibit a fronto-central midline predominance of power and minimum values over the temporal regions, while the highest values of the alpha band are seen at the parietal-occipital midline areas. The low level of sigma activity present in REM sleep peaks at centro-parietal midline sites. This frequency-specific regional pattern is substantially maintained in PAS-25 and PAS-50 nights.

The statistical maps of Fig. 4 reveal significant changes in the delta, theta, and alpha bands. Transcranial magnetic stimulation (comparisons between baseline vs. PAS-25 or PAS-50) lowered theta power in both PAS-25 and PAS-50 nights, in areas adjacent to the stimulated motor cortex: significant decreases were found at LMl, in PAS-50, and at LMp and at C3, in PAS-25. The decrease of EEG power at C3 scalp location extends to the alpha and sigma bands in the PAS-25 night. Here, too, the comparisons between PAS-25 vs. PAS-50 nights point to a pattern of specific EEG changes associated to synaptic potentiation. Significant changes affected the delta band. PAS-25 was associated with a higher delta power than PAS-50 at the F8 derivation. The congruence with the pattern found in NREM sleep is confirmed by the close-to-significance increase of power at Fp2 (p>0.02 and <0.05). The enhanced delta power at the right frontal sites coexists with a pattern of decreased activity in areas ipsilateral and contralateral to the hotspot of motor excitability, although no specific comparison was significant.

As a preliminary synthesis, magnetic stimulation, regardless of LTP-like changes, induced some EEG topographical changes in both REM and NREM sleep: the midline fronto-central sites increased delta power in both states, the occipital areas show an increased theta and alpha activity in NREM sleep, and some sites adjacent to the stimulated motor cortex show a decreased theta and alpha activity in REM sleep.

**Figure 5:**
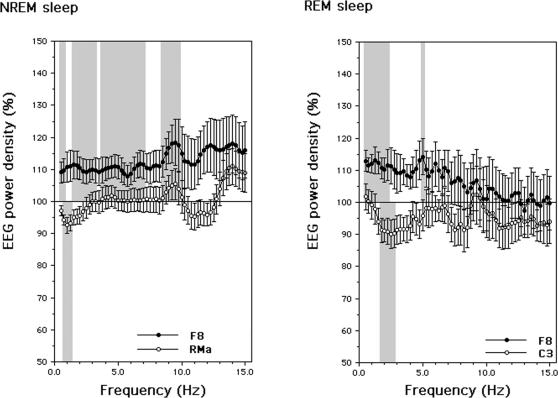
Frequency-specific changes of EEG power density at scalp locations showing the largest modifications in NREM (left panel) and REM (right panel) sleep in the PAS-25 vs. PAS-50 night comparison. EEG power during the PAS-25 night in the 0.50–14.75 Hz frequency range is expressed as percentage of the corresponding power during the PAS-50 night. Shaded areas point to significant comparisons between the PAS-25 vs. PAS-50 night.

In line with the hypothesis, SWA is affected by the waking potentiation of synaptic transmission in the motor cortex following conditioning sensory stimulation at the 25-msec interval, but with a complex pattern of changes. *A priori* unpredicted, the SWA enhancement in NREM is paralleled by a very similar increase in REM sleep. Slow-wave activity increases in right prefrontal sites and decreases at C3 and at sites contralateral to the stimulated left motor cortex. On the other hand, the theta power increases in left frontal sites, but only in NREM sleep. Frequency-specific changes at the scalp locations showing the largest modifications in NREM and REM sleep, share an enhancement of SWA in the PAS-25 night (Figure 5). This effect, consequent to the waking potentiation, persists up to 10 Hz in NREM encompassing the delta, theta and alpha bands. On the other hand, the unexpected fronto-central decrease of power in areas ipsilateral and contralateral to the excitability hotspot is frequency-specific, i.e. it is limited to certain frequencies in the delta range in both REM and NREM sleep.

**Figure 6:**
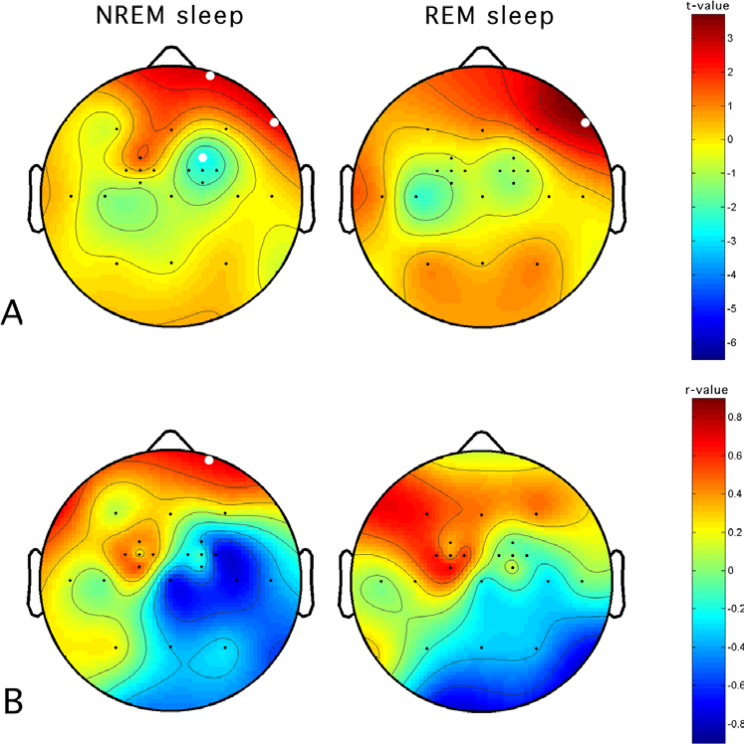
Magnitude of the changes in slow-wave activity (SWA) during NREM sleep predicts the increase in motor evoked potentials in the PAS-25 vs. PAS-50 condition, at each electrode. Panel A. Topographic statistical distribution of the change in SWA (0.50–4.75 Hz) by comparing NREM (left panel) and REM sleep (right panel) EEG during PAS-25 vs. PAS-50. Values are expressed in terms of t-values. Filled white circles indicate electrodes showing a significant difference (*t*≤2.82). Panel B. Topographic distribution of non-parametric correlations between the SWA change and the increase in motor evoked potentials (MEP) consequent to the PAS-25 protocol. Filled white circle indicate the electrode with a significant difference in Panel A also showing a significant MEP-SWA correlation (r = 0.72; p<0.05).

To give further support to the hypothesis of a causal link between the increase in motor evoked potentials during wakefulness, induced by LTP-like stimulation, and the magnitude of the SWA changes during sleep, we examined whether changes in SWA topography both in NREM and REM sleep are predicted by the increase of motor evoked potentials in PAS-25. Hence, we calculated correlation coefficients for the changes of motor evoked potentials in relation to the local SWA. Local changes of SWA were expressed in terms of SWA power in the night after LTP-like stimulation (PAS-25) relative to SWA power in the night after control stimulation (PAS-50). It emerges that changes of motor evoked potentials predict local changes of SWA in both NREM and REM sleep. Figure 6 illustrates topographically across all electrodes the correlation values (panel B) between the change in motor evoked potentials and the change in SWA during subsequent sleep. In both NREM and REM sleep correlation maps, a clear dissociation between areas with positive and negative values is visible: the waking potentiation of motor evoked potentials is associated with an increased SWA in prefrontal sites and a decreased SWA in more posterior sites. The correlation maps show more similarities with the maps of SWA changes in NREM than in REM sleep (panel A). The probability of correlations for those sites affected by the waking potentiation (three electrodes in NREM and one in REM sleep showing significant changes in the PAS-25 vs. PAS-50 comparisons) points to a significant positive relation only for the Fp2 electrode (r = 0.72; p<0.05). Therefore, the increase in motor evoked potentials evoked by magnetic stimulation during wakefulness predicted the magnitude of the increase in SWA during NREM sleep at the above-mentioned frontopolar derivation.

#### Time course of EEG changes

**Figure 7:**
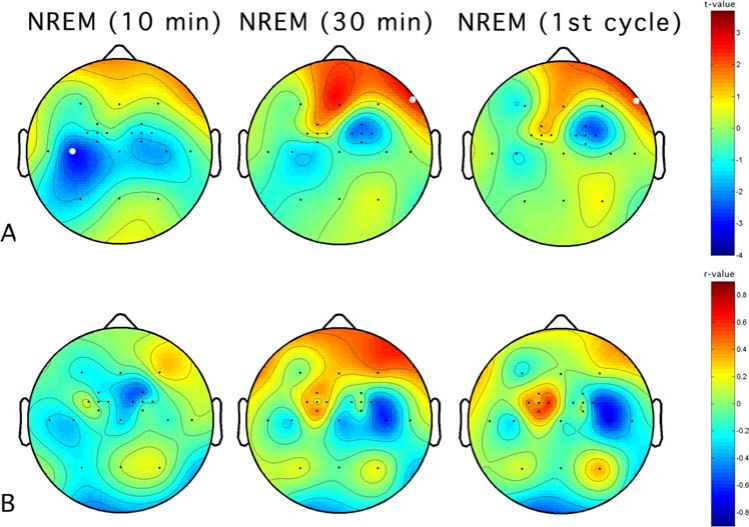
Time course of the relationship between the increase motor evoked potentials (MEP) in the protocol with paired associative stimulation at 25-ms (PAS-25) during wakefulness and the magnitude of the changes in slow-wave activity during sleep, at specific time intervals from sleep onset. Panel A. Topographic statistical distribution of the change in slow-wave activity (0.50–4.75 Hz) by comparing PAS-25 vs. PAS-50 during the first 10 min (left side), the first 30 min (middle), and the first NREM cycle (right side). Values are expressed in terms of t-values. Filled white circles indicate electrodes showing a significant difference (*t*≤2.82). Panel B. Topographic distribution of non-parametric correlations between the slow-wave activity change and the increase in motor evoked potentials consequent to the paired associative stimulation at 25-ms.

In view of a link between the increase in motor evoked potentials during wakefulness and the magnitude of the changes in SWA during sleep, the maps of SWA in the PAS-25 and PAS-50 nights at specific time intervals from sleep onset were analyzed and statistically compared taking into account the first 10 min of NREM, the first 30 min, and the whole first NREM cycle (Figure 7A). The maps of SWA power suggest a different time course for the incremental and decremental phenomena. While the maxima of the fronto-central decrease of SWA during PAS-25 compared to PAS-50 were found in the first 10 min of NREM sleep (significant at C3), the maxima of the increased SWA were reached during the remaining part of the first NREM cycle (significant at F8). Interestingly, the maxima of the fronto-central decrease of SWA were more pronounced in the stimulated hemisphere during the first 10 min, and then migrated to the contralateral hemisphere. The correlational maps at the same time intervals (Figure 7B) roughly reproduce changes in EEG power.

## Discussion

The present study was aimed to compare EEG topographical changes during sleep immediately following a presleep synaptic potentiation induced by an LTP-like paradigm (PAS-25) with EEG changes during sleep following a presleep control condition not affecting potentiation (PAS-50). The efficacy of PAS-25 in inducing LTP-like effects should be indexed by significant increases of motor evoked potentials in the stimulated *abductor pollicis brevis* muscle, while the specificity of this effect should be demonstrated by the non-significant variation of motor evoked potentials in other muscles. The *a priori* hypothesis predicted a localized enhancement of SWA after the waking PAS-25 paradigm, but not after the PAS-50.

The experimental manipulation of motor cortex excitability obtained in the present protocol with paired associative stimulation induced a 48% increase in motor evoked potentials. As expected, this increase was obtained only with an interval of 25 ms between the peripheral nerve electric stimulus and the transcranial magnetic pulse to the brain. On the other hand, an interval of 50 ms did not induce significant changes. More than any kind of sham stimulation, the PAS-50 condition represents a real control when investigating the subsequent LTP-like effects within the frame of the paired associative stimulation protocol. The specificity of this effect is further demonstrated by the non-significant variation of motor evoked potentials in the *abductor digiti minimi* muscle, innervated by a different nerve (ulnar) respect to that one for the conditioning stimulation (median). Therefore, the paired associative stimulation is effective and specific in increasing amplitudes of muscular responses within the territory of median nerve innervation, i.e. changes that are likely produced by an LTP-like mechanism at the contralateral sensorimotor primary cortices [14]–[16], [20], [21].

The macrostructural aspects of sleep were not affected by the experimental manipulation. On the other hand, a composite pattern of changes was found in EEG power maps of both NREM and REM sleep. A distinction should be made between *nonspecific* and *specific* effects. *Nonspecific* effects refer to changes in EEG power that were found when comparing post-stimulation sleep with baseline sleep, without any possibility of disentangling the effects due to paired stimulation *per se* from the specific LTP-like mechanisms. One or more aspects of the paired associative stimulation procedure evidently induced changes of EEG power in both NREM and REM sleep, and these changes were maximal for SWA in the fronto-central midline sites. Therefore, any of the following procedural aspects could have affected EEG power during the following sleep: the assessment of individual motor thresholds, the measurement of motor evoked potentials in response to magnetic pulses at 130% of the individual motor threshold, the delivery of electrical stimuli to the right median nerve, the re-assessment of motor threshold and of motor evoked potentials.

The *specific* effects, i.e. those directly related to the LTP-like mechanisms induced by the paired associative stimulation at 25 ms procedure, will be discussed in more detail below.

### EEG changes during the whole sleep night

This is the first study examining the effects of local (cortical) synaptic changes during waking on local EEG changes during the subsequent full night of sleep. In fact, effects of a learning task involving specific brain areas [11], of an arm immobilization [13], and of 5-Hz repetitive transcranial magnetic stimulation that induced a localized potentiation in the motor cortex [12] have been investigated only during the first NREM sleep episode. This was due to the intrinsic technical limitations of sleep recording for the whole night with high-density EEG. The current study is thus the first evaluation of the effects of a waking synaptic potentiation on EEG power of NREM/REM sleep cycles subsequent to the first one. The full post-potentiation night of sleep is characterized by two main changes, mostly shared by NREM and REM stages: frontal and prefrontal areas show an increased SWA, while areas ipsilateral and contralateral to the stimulated motor cortex show a decreased activity in the same frequency range. This pattern of enhancements and reductions is partly confirmed also for the theta frequency.

The effects on REM sleep could be considered particularly unexpected. Indeed, the synaptic homeostasis hypothesis [10] implicitly associates the extent of plastic changes in local cortical circuits during waking to the homeostatic changes of SWA during NREM sleep. In fact, there are two different but concurrent explanations for the present findings.

First, a recent study showed that electrophysiological indicators of synaptic strength, associated with high and low sleep pressure, as indicated by time spent awake, change in a similar manner in active wakefulness, NREM sleep and REM sleep [22]. In other words, a measure of synaptic efficacy, expressed by the slope of cortical evoked responses, was differentially responsive to high and low sleep-pressure conditions. This effect was found regardless of the behavioural state in which it was collected, i.e. active wakefulness, quiet wakefulness, NREM sleep and REM sleep.

Second, REM sleep and NREM sleep have some specific homeostatic rules in common, such as the increased SWA in the recovery after selective and partial sleep deprivation [9], [23]. Hence, the link between plastic changes in specific cortical circuits during waking and the subsequent homeostatic changes of sleep may be independent of the neurophysiological and neuromodulatory differences between NREM and REM sleep.

### Regional specificity

Neurophysiological mechanisms and cortical circuits involved in protocols with paired associative stimulation are still relatively unknown, and inferences about topographical mapping of induced changes in cortical excitability are limited. The LTP- and LTD-like effects that are induced in humans using paired associative stimulation procedures probably spread to different cortical sites. In fact, a variety of protocols that have induced LTP-like effects have shown a spreading from the motor to the somatosensory or to the frontal cortices [24]–[28], suggesting that cortical sites connected with M1 may have been influenced by the LTP- and LTD-like conditioning. An LTP-like effect has been directly induced by a repetitive magnetic stimulation at 5-Hz that increases coregistrated cortical responses to single magnetic pulses, as measured by combined high-density EEG before and after applying the conditioning stimulation [28]. This protocol was then associated to a local increase in SWA during the first 30-min of NREM sleep. Such SWA enhancement was positively correlated with the magnitude of waking potentiation [12]. However, the relationship between synaptic plasticity and sleep homeostasis was found in the premotor area (BA6), without appreciable changes in the motor cortex itself.

In our study, too, the increase of SWA is maximal in regions different from the stimulated M1. More specifically, the largest effect was found at the Fp2 and F8 sites during NREM sleep, and it extends to left premotor sites (LMa). LTP-like effects seem thus liable to affect cortical sites different from the conditioned one, but highly connected to it. Accordingly, protocols inducing LTP- and LTD-like effects have been recently found to be associated to an increase of SWA within the first hour of NREM sleep only in the hemisphere contralateral to the potentiated one. These protocols also induced an increase of 10–13 Hz EEG activity in both hemispheres [29], with no effect on the stimulated M1.

The SWA enhancement over frontopolar and prefrontal regions coexists with a specific topographical pattern of decreased SWA in areas ipsilateral and contralateral to the stimulated left motor cortex. Although not significant, a centro-parietal pattern (ipsilateral and contralateral to the stimulated M1) of decreased SWA was also found by Huber et al. [12]. Actually, several studies using protocols with paired associative stimulation over the motor or somatosensory cortex challenge the concept of an univocal correspondence between functional gain and enhancement of synaptic efficacy [30]. Likely, neural reorganization consequent to the stimulation of the motor cortex in humans influences neural processing not only at the site of stimulation but also at distant, interconnected sites [24]–[27]. After a cortical response was elicited by magnetic stimulation of the hand area, high-density EEG recordings showed activation of the adjacent ipsilateral motor area, which then spread to homologous regions of the opposite hemisphere [31], [32].

Moreover, experimental protocols inducing LTP-like changes with transcranial magnetic stimulation determine the simultaneous activation of both excitatory and inhibitory neurons at the site of stimulation [33]. Empirically, scalp maps of the differences between somatosensory evoked potentials at 35 ms after the median nerve stimulation, recorded before and after paired associative stimulation, show a midline frontal and prefrontal increase coexisting with a left fronto-central decrease [30], comparable to our findings in sleep.

Indeed, any new learning is based on both excitatory and inhibitory activations of different brain circuits. It has been demonstrated that learning involves specific enhancements of synaptic strength, induced by the activity-dependent coincident firing of pre-and post-synaptic neurons [34], [35]. Coincident firing of pre-and post-synaptic neurons at appropriate intervals is associated to LTP and LTD, which induce respectively potentiation and depression of synaptic transmission in converging pathways [36]–[38].

The spatial pattern observed in both NREM and REM sleep is reminiscent, in some respects, of the perceptual “excitatory centre–inhibitory surround” organization encountered in cortical physiology [39]. Probably, the selective stabilization of learning-related neuronal connections and, consequently, the memory formation need a focal excitability increase of some neuronal networks, enhancing the excitability and synaptic strength of learning-related neuronal connections, but inhibiting the excitability of others. The co-existence of regions with enhanced or decreased SWA suggests that induced LTP-like plasticity may display a spatial organization in multiple systems, sculpting a kind of centre–surround organization.

### After-effects of waking potentiation

Previous studies evaluating the effects on subsequent sleep of a learning task involving specific brain areas [11], of an arm immobilization [13], and repetitive magnetic stimulation at 5-Hz inducing a localized potentiation in the motor cortex excitability [12], took into account only the first NREM sleep episode. They found a progressive dissipation of the effects consequent to the waking synaptic potentiation, that was completed within 20- to 40-min [11]–[13]. Also the most recent study correlating interindividual differences in the magnitude of a cortical potentiation and suppression with EEG activity during sleep in the 10–13 Hz frequency range, found that association within the first 60 min of sleep [29].

Our analyses of the time course of SWA changes after paired associative stimulation at 25 ms only partly support this phenomenon during the first NREM cycle. A close inspection of Fig. 7 actually suggests that the first minutes of sleep are mostly characterized by a decreased SWA at the electrodes adjacent to the stimulated M1, while the expected increase of SWA is discernible only after 30 minutes from sleep onset. Then, this increased SWA remains stable in the entire first sleep cycle, and shows the same pattern of topographical changes seen during the whole night. These results are suggestive of different potentiation mechanisms affecting SWA subsequent to paired associative stimulation at 25 ms, that is short- and long-term plasticity. If long-term plasticity refers to persistent modifications in synaptic efficacy, short-term plasticity reflects the reversible modulation of synaptic strength in response to varying presynaptic stimuli. In rats, short-term plasticity effects have also been observed after paired pulse stimulation of the white matter projecting to the motor cortex [40]. In humans, the existence of changes in short-term plasticity has been suggested after paired associative stimulation over the motor cortex [41]. Although speculative, the pattern of changes observed within the first 10 min from sleep onset points to different influences of plasticity mechanisms on subsequent SWA, as a function of the time elapsed from waking potentiation. In other words, the pattern of decreased SWA in the left somatosensory cortical regions may be associated to presynaptic changes in short-term plasticity consequent to the recurrent stimulation of the right median nerve during the paired-associative protocol. An independence of changes within the first 10 min from those occurring in the last part of the first sleep cycle seems indirectly confirmed by the lack of any consistent relationship between the extent of changes in SWA and the increase in the amplitude of motor evoked potentials in paired associative stimulation at 25 ms (Figure 7, panel B).

### Conclusions

The present findings further corroborate the notion that changes in synaptic strength, presumably induced by LTP-like protocols in humans, lead to changes in local sleep regulation. Specifically, the positive correlation between the spatial pattern of SWA changes and the magnitude of the plastic changes associated to paired associative stimulation at 25 ms in the whole night's sleep supports the hypothesis of a relationship between LTP-like changes and sleep need, the latter expressed by the regional increase of SWA. Moreover, such SWA increase suggests that paired associative stimulation leaves long-term influences. Somehow, this finding strengthens the notion that sleep is characterized by local changes that could be related to long-lasting potentiation (or depression) of specific neural pathways due to waking activities, i.e. the synaptic history of excitatory and inhibitory activations of different circuits.

Undoubtedly, there are some limitations of our findings. The spread from the stimulated motor cortex to prefrontal regions and the simultaneous activation of both excitatory and inhibitory circuits remain speculative interpretations. However, a better knowledge of the specific neurophysiological mechanisms and cortical circuits involved in the PAS protocol is needed. In fact, a precise mapping of the actual changes in cortical excitability induced by paired associative stimulation is, at present, not available. Moreover, there is only indirect empirical evidence that paired associative stimulation after-effects rely on mechanisms similar to those of synaptic plasticity studied at the cellular level [14], [16], [42], [43]. These issues should be clarified in the near future.

## Materials and Methods

### Subjects

Ten right-handed healthy experimental subjects (5 males and 5 females, mean age = 23.00±2.21) were selected from a university student population with no personal or family history of epilepsy or other neurological or psychiatric disease. Further requirements for inclusion were: normal sleep duration (habitual sleep time: 24.00–8.00±1 hr) and schedule, no daytime nap habits, no excessive daytime sleepiness, no other sleep, medical or psychiatric disorder, as assessed by a one-week sleep log and by a clinical interview. Participants were required to avoid napping throughout the experiment; compliance was controlled by actigraphic recordings (AMI Mini motion logger). Drugs and caffeinated beverages were not allowed.

All subjects gave their written informed consent. The study was approved by the Institutional Ethics Committee of the Department of Psychology of University of Rome “Sapienza” and was conducted in accordance with the Declaration of Helsinki.

### Materials

#### Transcranial magnetic brain stimulation and electrical nerve stimulation

The study used a Magstim 200 Mono Pulse connected to a Bistim module and a figure-of-eight coil with an external diameter of 9 cm (Magstim Company Limited, UK). The peak magnetic field produced by such a coil is 2.0 T. Electrical stimulation was delivered using a bipolar electrode (cathode proximal) placed on the right median nerve at the level of the wrist, connected to an electromyograph (Myto, EBNeuro, Italy).

Motor evoked potentials (MEPs) were obtained from the right *abductor pollicis brevis* and *abductor digiti minimi* muscles by positioning two Ag-AgCl surface cup electrodes of 9 mm diameter with the active electrode on the muscle belly and the reference electrode on the metacarpophalangeal joint. Skin-electrode conductance was kept to <10 KΩ. The electromyographic signals were band-pass filtered between 1 and 1 K Hz, recorded using an electromyogram-dedicated software (Myto, EBNeuro, Italy), and stored in a computer for off-line analysis.

#### Polysomnographic recordings

A Micromed HandyEEG Sistem Plus polygraph was used for polygraphic recordings. EEG signals were high pass filtered with a time constant of 0.3 s and low pass filtered at 30 Hz. The nineteen unipolar EEG derivations of the international 10–20 system (Fp1, Fp2, F7, F8, F3, F4, Fz, C4, C3, Cz, P3, P4, Pz, T4, T6, T3, T5, O1, O2) were recorded from scalp electrodes with averaged mastoid reference. Ten additional unipolar EEG derivations were recorded. One electrode was positioned exactly over the *individual* optimal site of stimulation (“hotspot”), and other four electrodes were placed around this point in each orthogonal direction at a distance of 1.5 cm. The hotspot for eliciting motor evoked potentials in the right *abductor pollicis brevis*, considered for this additional EEG montage, is very close and slightly posterior to the FC3 electrode [44] of the extended 10–20 system [45]. As a denotation, the four orthogonal positions will be indicated as “anterior to left motor cortex” (LMa), “medial to LM” (LMm), “posterior to LM” (LMp), and “lateral to LM (LMl)”. The same montage and denotation was also used for the contralateral hemisphere (RM, RMa, RMm, RMp, and RMl).

The submental electromyogram was recorded with a time constant of 0.03 s. Bipolar horizontal eye movements were recorded with a time constant of 1 s. The bipolar horizontal electrooculogram was recorded from electrodes placed about 1 cm from the medial and lateral canthi of the dominant eye. Impedance of these electrodes was kept below 5 KOhm.

### Experimental procedure

#### General procedure

Each subject participated in the study across 4 consecutive nights. The sleep recordings, carried out in a sound-proof, temperature-controlled room, were scheduled in the first night (adaptation), in the second night (baseline sleep), and in the third and fourth nights (post-PAS sleep). In one night subjects were submitted to a paired associative stimulation at 25-ms intervals (PAS-25) and, in the other, to a stimulation at 50-ms intervals (PAS-50). The sequence of protocols at 25 ms and 50 ms was counterbalanced between subjects. Subjects were retested with the same protocol sequence immediately after final morning awakening, and any aspect of the morning protocol exactly matched the presleep protocol.

### Paired Associative Stimulation

Subjects were seated on a comfortable chair in a soundproof room and tested 90 min before the scheduled lights-off time. The coil was held tangentially to the skull, 45° from the midsagittal axis of the subject's head to stimulate fibers travelling horizontally with respect to the cortical surface [46]. The optimal site of stimulation (“hotspot”) for eliciting motor evoked potentials in the right *abductor pollicis brevis* was chosen by positioning the coil approximately over the central sulcus and moving it on the scalp in 0.5 cm steps on the primary motor area (M1) of the left cortex. The hotspot was marked on the scalp with a soft-tip pen.

Motor threshold was assessed at rest over the hot spot of the *abductor pollicis brevis* muscle and corresponded to the lower intensity of stimulation able to produce at least 50 μV in the relaxed muscle in at least five out ten consecutive stimulations [47]. Stimulation started at suprathreshold intensity (about 60% of stimulator output) and was decreased in steps of 1–2% of the stimulator output [46]. Then the intensity of stimulation necessary to elicit reliable motor evoked potentials in 90% of a cascade of 10 stimuli was assessed. This intensity was 130% of the individual motor threshold [21] (mean intensity output in PAS-25 = 53.2±2.71; mean intensity output in PAS-50 = 53.2±2.60) and it was kept constant throughout the session.

Paired associative stimulation (PAS) consisted of 140 pairs of stimuli delivered in 23 min. The magnetic stimulus, delivered on the optimal site for the *abductor pollicis brevis* muscle on the left cortex, was preceded by an electrical stimulus delivered to the right median nerve at wrist level, with an interval of 25 ms [14], [15]. The electrical stimulus lasted 200 μs and had an intensity of three times the perceptual threshold (averaged intensity of electric stimulation in PAS-25 = 4.71±0.15 mA, averaged intensity of electric stimulation in PAS-50 = 4.65±0.18 mA; two-tailed t-test = 0.26, p = 0.80).

The repetition rate of paired stimulation was 0.1 Hz [48]. During PAS, subjects were asked to watch their own right hand, since this condition has been demonstrated to give the maximal plasticity induced by PAS [49]. The experimental session lasted about 90 min. Before and after PAS, motor thresholds of the *abductor pollicis brevis* muscle were assessed and twenty motor evoked potentials elicited by single magnetic pulses were collected with a stimulation rate of 0.20 Hz.

Subjects were required to keep their right arm and hand completely relaxed. Muscle relaxation was monitored by giving subjects a visual feedback of their electromyogram, and trials contaminated with voluntary electromyogram activity were discarded from further analysis.

### Sleep study

The subjects' sleep was undisturbed in all three nights, started at 24.00 hours, and ended after approximately 8 hours of accumulated sleep (as online visually checked by expert sleep researchers). All awakenings were scheduled from stage 2 sleep. Just after awakening from nights 3 and 4, each subject participated in a retest session of paired associative stimulation (data not shown).

A diagram of the complete experimental protocol is reported in Figure 1.

### Data analysis

#### Transcranial magnetic stimulation measures

##### Motor thresholds

Resting motor thresholds were expressed as the magnetic stimulator output ± S.E.M. Motor thresholds were analyzed by a within-subjects analysis of variance design Treatment (pre-PAS vs. post-PAS) × Night (PAS-25 vs. PAS-50). This analysis aimed to evaluate differences in motor thresholds after the intervention and the stability of this measure between the two experimental conditions.

##### Paired associative stimulation

The dependent variable was amplitude of motor evoked potentials recorded in the target muscle (*abductor pollicis brevis*) and in the control muscle (*abductor digiti minimi*), before and after the paired associative stimulation (respectively, *pre-PAS* and *post-PAS*). Amplitude of motor evoked potentials was measured peak-to-peak, and then log-transformed before any statistical procedure, in order to obtain a better approximation to gaussianity and a higher homoschedasticity.

To test changes in motor evoked potentials of the *abductor pollicis brevis* muscle after PAS with different intervals, a within-subjects analysis of variance, Treatment (pre-PAS vs. post-PAS) × Night (PAS-25 vs. PAS-50) was performed. The same analysis was carried out on motor evoked potentials of the *abductor digiti minimi* muscle to assess the specificity of the treatment effect. The level of significance was set at p≤0.05.

### EEG measures

#### Polysomnographic measures

Cz EEG (Cz-A1), electromyogram, and electrooculogram were used to visually score sleep stages in 20 sec epochs, according to the standard criteria [29] by an experimenter who was blind with respect to the treatment condition. With regard to slow-wave sleep scoring, the >75 μV amplitude criterion was strictly followed [50].

The following were considered as dependent variables: sleep onset latency, defined as stage 1 latency; stage 2 latency; total sleep time (TST), defined as the sum of time spent in stage 1, stage 2, slow-wave sleep and REM; total bedtime (TBT), sleep efficiency index (SEI = TST/TBT×100), number of awakenings, wakefulness after sleep onset, percentage of each sleep stage and percentage of arousals. The polysomnographic EEG measures were submitted to one-way repeated measure analyses of variance, comparing baseline (BSL), PAS-50, and PAS-25 night.

#### Quantitative analysis of EEG

The polygraphic signals (29 EEG channels, electromyogram, and electrooculogram) were analog to digital converted on-line with a sampling rate of 128 Hz and stored on the disk of a personal computer. Artifacts were excluded off-line on a 20-sec basis by visual inspection; as regards REM sleep, only tonic periods were included in the analyses, to avoid artifacts from rapid eye movements on EEG power. Power spectra of the 29 derivations were computed by a Fast Fourier Transform routine for consecutive 4 sec epochs, resulting in a frequency resolution of 0.25 Hz. Values above 25 Hz were not used in the analysis. By collapsing four adjacent 0.25-Hz bins (1–25 Hz), the data were reduced to a 1-Hz bin width. The only exception was the 0.5–1.0 Hz bin, for which two adjacent 0.25-Hz bins were collapsed.

A further data reduction of power spectra was achieved by averaging 15 consecutive 4-sec epochs to yield a 60-sec spectrum. As a result, this spectrum included three consecutive 20-sec visually scored epochs. Power spectra were calculated separately for NREM (stage 2+3+4) and REM sleep. The bins are referred to and plotted in this study by the highest frequency included (e.g., the 3-Hz bin refers to the averaged values of the following bin intervals: 2.00–2.25, 2.25–2.50, 2.50–2.75 and 2.75–3.00 Hz). Bins were then collapsed to obtain the following EEG bands: delta (0.5–4.75 Hz), theta (5.00–7.75 Hz), alpha (8.00–11.75 Hz), sigma (12.00–14.75 Hz).

EEG power values for each frequency band were considered as dependent measures. Values were normalized by total power of the recording, color coded, plotted at the corresponding position on the planar projection of the scalp surface, and interpolated (biharmonic spline) between electrodes.

EEG power maps were computed separately for the following nights/conditions: baseline (*BSL*), PAS-25 treatment (*PAS-25*), and PAS-50 treatment (*PAS-50*).

Statistical comparisons between PAS-25 and PAS-50 vs. BSL point to aspecific effects of TMS on sleep, while comparisons between PAS-25 vs. PAS-50 point to specific effects of LTP-like changes on sleep. To correct for multiple comparisons, the Bonferroni correction was applied. Considering the mean correlation between the variables (r = 0.73), the alpha level was then adjusted to ≤0.02 (t≥2.82).
